# Bee sting presumed endophthalmitis: a devastating ocular outcome

**DOI:** 10.1186/s40942-021-00320-w

**Published:** 2021-09-06

**Authors:** Mohammed Al Amry, Huda Al Ghadeer, Ahmed R. Al Gethami

**Affiliations:** 1 Emergency Department, King Khaled Eye Specialist Hospital, Riyadh, Saudi Arabia; 2 Retina Department, King Abdullah Medical City, PO Box 24246, Makkah, 21955 Kingdom of Saudi Arabia

**Keywords:** Bee, Endophthalmitis, Injury, Ocular

## Abstract

**Background:**

To report a rare case of bee sting presumed endophthalmitis that resulted in a devastating ocular outcome.

**Case presentation:**

A 43-year-old patient presented 24 h after bee sting ocular injury in his left eye. He had a mild inflammatory sign at presentation, and he underwent surgical exploration to rule out a scleral defect, which revealed a sealed defect. During his hospital course, he developed signs of endophthalmitis 48 h following trauma for which he received vitreous tap and intravitreal antibiotic. Microbial culture revealed gram-negative rods, *Pseudomonas aeruginosa*, and *Aeromonas veronii*. Condition escalated to reach the panophthalmitis stage and cellulitis like picture with visual acuity of no light perception. Visual evoked response (VER) demonstrated a flat response. Infection was controlled by evisceration of the globe at the end as salvage therapy against the spreading of infection

**Conclusions:**

Bee sting ocular injury is an exceedingly rare type of ocular trauma. Concomitant infection can happen, and severity depends on the pathogen involved. It is crucial to have insight and start appropriate treatment based on to the patient presentation.

## Introduction

Bee bites can cause ocular injuries [1–4]. Local and systemic effects that can happen is related to toxins released from venom contained in the sting, the eye-related injuries reported are conjunctivitis, corneal infiltrates, cataract formation, iritis, hyphema, lens subluxation, and optic nerve damage secondary to glaucoma [5]. Corneal injury is the most commonly reported ocular injury [1–5]. Optic neuropathy, ciliochoroidal detachment and endophthalmitis have also been reported in very few reports [6–8]. In this report, we describe a rare case of endophthalmitis secondary to bee sting with devastating ocular outcome.

Although patient’s identity has not been revealed, consent was taken from the patient to publish the photographs.

## Case report

**Fig. 1:**
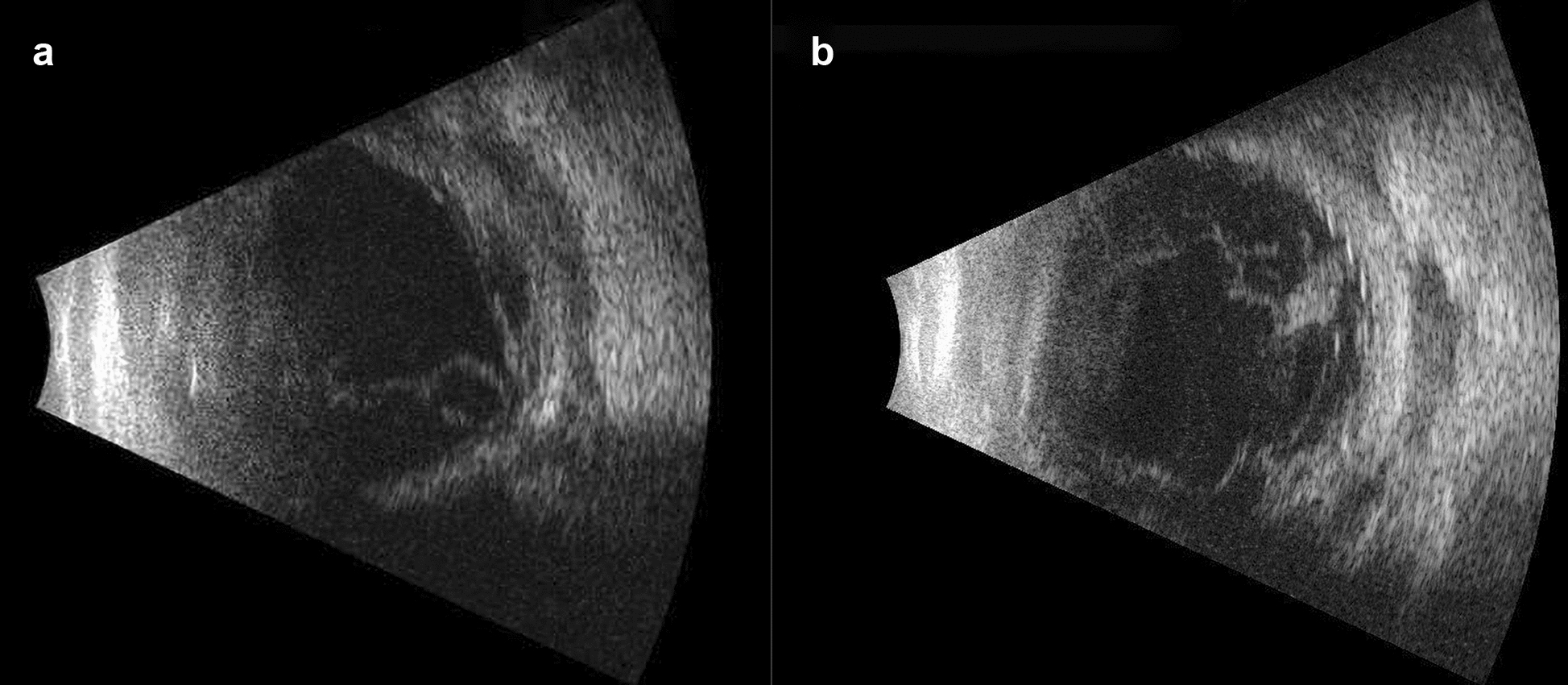
**A** B-scan Ultrasonography of left eye showing low reflectivity and thickening of the sclera at wound site. **B** B-scan Ultrasonography of left eye demonstrating more vitreous opacities with retina and choroid (RC) layer thickening suggesting endophthalmitis

A 43-year-old man presented to Emergency Room of King Khaled Eye Specialist Hospital with painful redness and loss of vision in his left eye following trauma by bee sting while he was outdoor one day prior to his presentation. He lost his right eye few years back due to open globe injury with multiple surgeries ending in blind painful eye. Apart from systemic hypertension his medical history was noncontributory. Ophthalmic examination indicated the best corrected visual acuity of poor light perception in the right eye (OD) and 20/200 in left eye (OS). (Documented previously to be 20/20). Intraocular pressure measured with applanation tonometry was within the normal range bilaterally. Examination of the right eye revealed two corneal sutures, no leak, deep and quite anterior chamber, posterior synechia, traumatic cataract and a hazy view of the fundus. Slit-lamp examination of the left eye indicated moderate conjunctiva injection, 1 mm × 1 mm conjunctival defect with inferior temporal subconjunctival hemorrhage. Clear cornea, no leak was observed. There was fibrinous reaction with +4 cells in the anterior chamber, early cataractous changes, vitreous hemorrhage, and hazy view to fundus. B-scan Ultrasonography revealed total closed funnel shape retinal detachment OD and low reflectivity, thickening of the sclera at wound site OS (Fig. 1A).

**Fig. 2:**
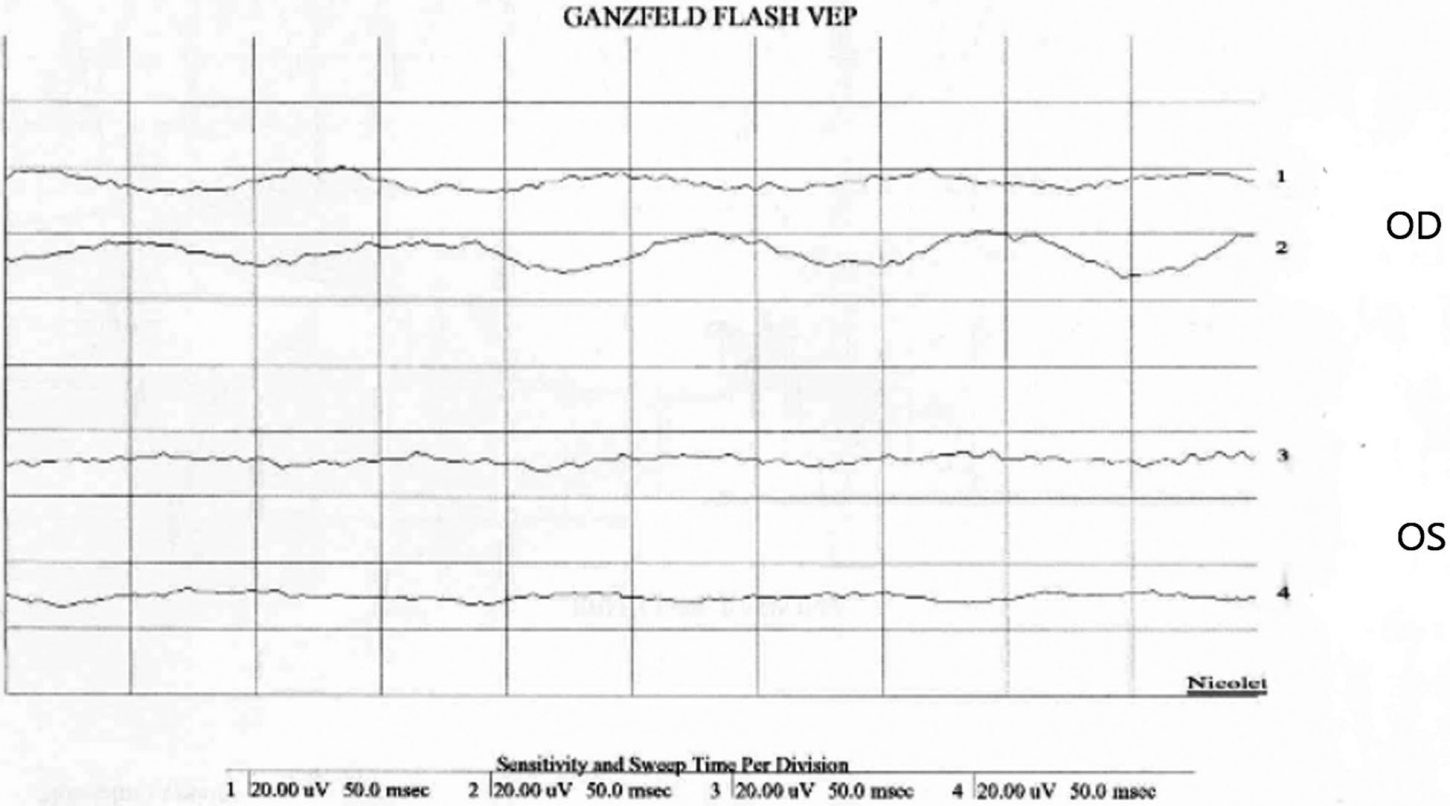
Visual evoked response (VER) demonstrating flat response in the left eye

**Fig. 3:**
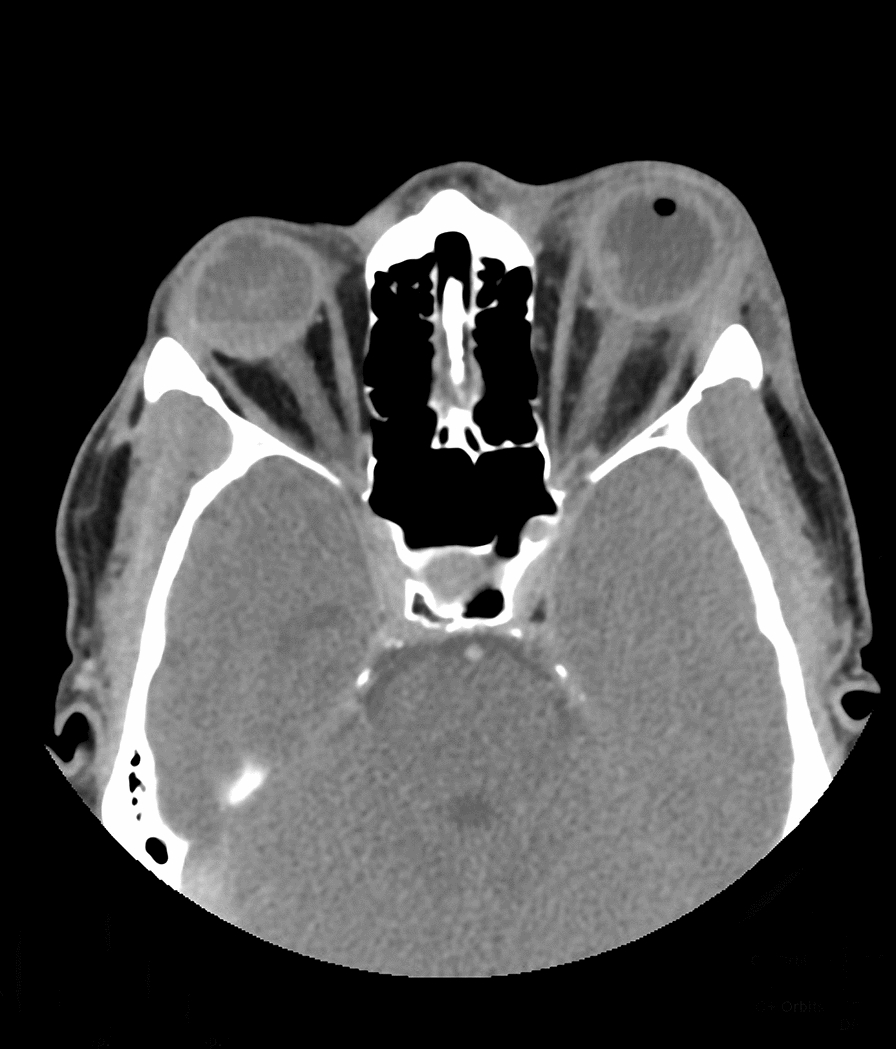
Computerized tomography (CT) of orbit revealed diffuse preseptal thickening with evidence of extension along the episcleral and proximal part of the optic nerve with infiltration of the tendinous space associated with diffuse thickening along the choroidoretinal thickening

Patient kept NPO, started on topical antibiotics (ofloxacin, 0.3% 6 times per day), IV antibiotics cefazoline 1 g every 8 h and gentamicin 80 mg every 8 h and prepared for immediate exploration in the operating room for the inaccessibility to perform full ocular examination and to exclude open globe injury. It was found that during surgery no clear defect or leak from sclera. He was kept on IV antibiotics. Few hours post operatively, patient developed significant corneal edema with high intraocular pressure (40 mm HG), that was managed by oral acetazolamide 250 mg four times daily, timolol maleate (timabak 0.50%) drop twice daily, latanoprost (xalatan) 0.005% once daily and brimonidine tartrate (alphagan-p-0.15%) twice daily. His course after surgery was deteriorating with poor vision (light perception), more pain, corneal edema, and development of hypopyon over 24 h period. Thirty-six hours post operatively his vision in the left eye drop to no light perception, another B-scan Ultrasonography examination (Fig. 1B) showed more vitreous opacities and retina and choroid (RC) layer thickening suggesting endophthalmitis, patient received intravitreal antibiotic of vancomycin 1 mg/0.1 ml and ceftazidime 2.25/0.1 ml and dexamethasone 0.4 mg/0.1 ml. Vitreous and anterior chamber tap was taken just before injecting intravitreal injection was positive for gram negative rods on gram stain and on culture *Pseudomonas aeruginosa* and *Aeromonas veronii* which also confirmed by another culture from corneal and at third day Visual evoked response (VER) demonstrated flat response (Fig. 2). The patient develop pain with eye movement which indicated worsening of his condition and transforming to panophthalmitis. Follow up B-scan showed vitreous opacities with vitreal membranes, localized retinal detachment detected around the equator with dense sub-retinal opacities, almost sealed scleral defect detected inferior temporal quadrants. Computerized tomography (CT) of orbit (Fig. 3) showed diffuse preseptal thickening with evidence of extension along the episcleral and proximal part of the optic nerve with haziness, and infiltration of the tendinous space with associated diffuse thickening along the choroidoretinal thickening. At this stage, as patient have endophthalmitis with scleritis and due to spread of infectious process, retina, and oculoplastic team advice for evisceration of globe. Evisceration done and patient continued his course of intravenous antibiotics without systemic spread of infection, and he was discharge in stable condition.

## Discussion

Toxic and immunologic responses are caused by inflammatory mediators and amines, enzymes, and polypeptide toxins in bee sting injuries. It can lead to both local reactions like erythema at the site of the sting and systemic reactions like severe anaphylaxis, demyelination, and shock. This can happen acutely or in delayed response [9]. Ocular changes involving the retina have been reported following a wasp and bee sting injury [2, 3, 10].

Nakatani et al. reported a case of panuveitis secondary to corneal wasp sting. They reported successful treatment by vitrectomy [3]. Rishi et al. reported a case of vitritis secondary to bee sting injury [10]. Narang et al. reported a case of bee sting induced *Aspergillus fumigatus* endophthalmitis and scleritis which was treated with antifungal agents topical, systemic, and intravitreal [3].

Our patient presented with subconjunctival hemorrhage at the site of the sting with mild anterior uveitis at presentation, he underwent exploration for suspicious scleral defect that was not found. After that, he progressed over short period to clinical picture of endophthalmitis with anterior uveitis, hypopyon, vitritis, and vitreal membranes to finally reaching panophthalmitis.

Our patient developed a conjunctival defect at the probable site of bee sting. The site of microperforation could have been inoculated with the bacteria carried by the bee. The histamine present in the bee sting leads to increase in the capillary permeability and can be the cause for the conjunctival hyperemia at the site of the sting. Bee venom may induce bacterial endophthalmitis concurrently with toxic injection. It is possible that the chemicals and bacteria present in the bee sting might have entered the vitreous cavity leading to vitritis and typical picture of endophthalmitis. Unlike Nakatani et al. case who presented with a picture of toxic anterior segment syndrome (TASS) and uveitis [3]. Knowing that infectious process is dependent on immunity of host and pathogen involved. It is important to consider incubation period of the pathogen, in our case symptoms of endophthalmitis started to appear at 48–72 h.

It is difficult to differentiate toxic sterile from infectious endophthalmitis, especially in cases of severe anterior inflammation induced by a toxic substance. Timing of presentation could give a hint to differentiation as our case escalated very fast as microbial endophthalmitis versus pure inflammatory process which may be a slower rate compared to infection. There was an absence of signs of sympathetic ophthalmia in the patient’s clinical presentations.

Gudiseva et al. reported a good outcome with oral steroid in addition to topical steroid and topical antibiotics especially for cases with severe anterior uveitis and corneal edema at presentation [5]. However, it is important to vigilant not to start oral steroid without ruling concomitant infectious process as it may worsen the condition. After controlling eye inflammation, functional testing can guide the management in patients with presumed optic neuropathy and endophthalmitis secondary to ocular bee sting. Ahmed et al. reported good final outcome in a case of optic neuropathy and presumed endophthalmitis, they used electro physiological testing to look for vision potential after controlling eye inflammation, restorative surgeries was taken and their patient reach vision of 20/80 from light perception [8].

## Conclusion

Bee sting ocular injury is very rare type of trauma. Concomitant infection can happen, and severity depend on pathogen involved. It is crucial to have insight and aggressive treatment according to patient presentation. If patient has an infective process, intravitreal antibiotics is important, otherwise, anti-inflammatory agent as steroid can play a role after ruling out infection.

## Abbreviations

OD (oculus dexter): Right eye
OS (oculus sinister): Left eye
NPO (nil per os): Nothing by mouth
RC: Retina choroid
VER: Visual evoked response
CT Scan: Computerized tomography
